# Rapid Secondary Recrystallization of the Goss Texture in Fe_81_Ga_19_ Sheets Using Nanosized NbC Particles

**DOI:** 10.3390/ma14143818

**Published:** 2021-07-08

**Authors:** Fan Lei, Yuhui Sha, Zhenghua He, Fang Zhang, Liang Zuo

**Affiliations:** 1 Key Laboratory for Anisotropy and Texture of Materials, Ministry of Education, Northeastern University, Shenyang 110819, China; leifan1028@163.com (F.L.); hezhenghua@mail.neu.edu.cn (Z.H.); zhangf@smm.neu.edu.cn (F.Z.); lzuo@mail.neu.edu.cn (L.Z.); 2 School of Materials Science and Engineering, Shenyang University of Technology, Shenyang 110870, China

**Keywords:** Fe–Ga alloy, Goss texture, secondary recrystallization, inhibitor, magnetostriction/thin sheet

## Abstract

Herein, a simple and efficient method is proposed for fabricating Fe_81_Ga_19_ alloy thin sheets with a high magnetostriction coefficient. Sharp Goss texture ({110}<001>) was successfully produced in the sheets by rapid secondary recrystallization induced by nanosized NbC particles at low temperatures. Numerous NbC precipitates (size ~90 nm) were obtained after hot rolling, intermediate annealing, and primary recrystallization annealing. The relatively higher quantity of nanosized NbC precipitates with 0.22 mol% resulted in finer and uniform grains (~10 μm) through thickness after primary recrystallization annealing. There was a slow coarsening of the NbC precipitates, from 104 nm to 130 nm, as the temperature rose from 850 °C to 900 °C in a pure nitrogen atmosphere, as well as a primary recrystallization textured by strong γ fibers with a peak at {111} <112> favoring the development of secondary recrystallization of Goss texture at a temperature of 850 °C. Matching of the appropriate inhibitor characteristics and primary recrystallization texture guaranteed rapid secondary recrystallization at temperatures lower than 950 °C. A high magnetostriction coefficient of 304 ppm was achieved for the Fe_81_Ga_19_ sheet after rapid secondary recrystallization.

## 1. Introduction

Fe–Ga alloy (galfenol) is an attractive magnetostrictive material for energy-harvesting devices, sensors and actuators. It has good properties, such as a very large magnetostriction coefficient, and excellent mechanical properties [1,2,3,4,5,6]. The alloy exhibits significant magnetocrystalline anisotropy and high electrical conductivity. Therefore, it is desirable to prepare thin sheets with strong η texture (<100>//rolling direction) by rolling methods. Secondary recrystallization, also termed abnormal grain growth (AGG), is the most effective way to realize sharp Goss ({110} 〈001〉) texture and larger magnetostriction coefficients in rolled Fe–Ga thin sheets by high-temperature annealing [7,8,9,10].

Na et al. reported that Goss grains with an area fraction of ~98% were obtained in rolled Fe–Ga sheets with a magnetostriction coefficient of 245–292 ppm using 1.0 mol% microsized NbC particles (1–5 µm) to inhibit the growth of matrix grains and annealing under an Ar and H_2_S atmosphere [11,12]. Yuan et al. [13,14] reported that strong Goss texture with a magnetostriction coefficient of 245 ppm was obtained in rolled columnar-grained Fe_83_Ga_17_ alloys by using 0.1 mol% microsized NbC particles, and the surface segregation of S was imperative for retarding the growth of surface grains as the annealing temperature rose to 1200 °C. Liu et al. [15,16] reported that the AGG of Goss grains was induced by nanosized NbC precipitate particles in rolled sharp <100>-oriented columnar-grained Fe_82_Ga_4.5_Al_13.5_ after annealing at 1085 °C. He et al. [17] suggested that the secondary recrystallization of the sharp Goss texture and a magnetostriction coefficient of 285 ppm were induced by the nanosized sulfides that were precipitated and dispersed, and secondary recrystallization was nearly completed at 1050 °C in Fe_81_Ga_19_ thin sheets.

When 1 mol% microsized NbC particles or 0.1 mol% microsized NbC precipitates are used as the inhibitor in an Fe–Ga thin sheet, high-temperature annealing and the surface-energy effect of a special annealing atmosphere are required for the development of secondary recrystallization of the sharp Goss orientation. When nanosized precipitates of NbC or sulfide are used as the inhibitor in an Fe–Ga thin sheet, a long annealing time (>10 h) and high temperature (>1050 °C) are necessary to ensure coarse inhibitors to induce the AGG of the Goss texture in the thin sheet. However, these conditions will reduce the magnetostriction coefficient of the thin sheets because of the volatilization loss of Ga element after annealing at high temperature for a long time [18]. Therefore, these challenges provided the motivation to synthesize a giant magnetostrictive Fe–Ga thin sheet by rapid secondary recrystallization at lower temperatures without utilizing the surface-energy effect.

In general, AGG is more likely to develop earlier and faster with finer primarily recrystallized grains, appropriate grain-boundary conditions, and inhibitor characteristics that follow the modified Hillert equation [19]. The growth of surface grains, in particular, plays a vital role in the process of secondary recrystallization, except for the internal matrix grains. For secondary recrystallization to occur, the inhibitors should apply sufficient pinning force on the matrix grains, and preferred growth for Goss grains by weakened pinning force from coarsening of inhibitors. Precipitates sized 30–100 nm can impose a strong inhibiting force on the primary grains, and the inhibiting force is clearly lower when the size is beyond this range. However, complete secondary recrystallization cannot be induced only by 0.1 mol% nanosized NbC precipitates in an Fe–Ga thin sheet; strong Goss texture with size advantage in primary recrystallization realized by initial directional solidification is necessary [13,14]. The sulfide precipitates sized 30–50 nm require a higher temperature and longer time to coarsen, and provide the driving force necessary for the AGG of Goss grains [17]. The matching of primary recrystallization microstructure, texture, and characteristics of precipitates plays a vital role in the process of secondary recrystallization, which have not been studied in detail. Therefore, to achieve rapid secondary recrystallization at low temperatures by traditional rolling and annealing, the characteristics of precipitates and primary recrystallization should be reasonably regulated.

In this study, 0.22 mol% nanosized NbC particles precipitated during rolling and annealing were used to enhance the inhibitor in an Fe_81_Ga_19_ alloy thin sheet. The rapid secondary recrystallization of the sharp Goss orientation and a magnetostriction coefficient of 304 ppm were successfully achieved at 950 °C without any special atmosphere. The inhibitor, microstructure, and texture evolution were explored to clarify the development of rapid secondary recrystallization at a low temperature in the thin sheet.

## 2. Experimental Procedure

The Fe_81_Ga_19_ + 0.22 mol% NbC alloy ingots were prepared from pure Fe (99.99%) and Ga (99.999%) and master alloys of Nb–Fe and Fe–C by arc melting. The ingots were reheated to 1250 °C and hot rolled (indicated as HR) to 2.4 mm with a finishing temperature of 800 °C. Then, the hot-rolled bands were first-stage cold rolled (indicated as 1CR) to 1 mm at 200 °C with a reduction of 58%. Afterwards, intermediate annealing (indicated as IA) was performed at 975 °C for 8 min. Subsequently, the annealed sheets were further cold rolled (indicated as 2CR) to 0.25 mm at 200 °C with a reduction of 75%. The cold-rolled sheets were annealed (indicated as PR) at 850 °C for 5 min to achieve primary recrystallization. Finally, secondary recrystallization annealing was performed as the temperature was raised from 800 °C to 950 °C at a heating rate of 15 °C/h in a flowing N_2_ atmosphere. The inhibitor, microstructure, and texture evolution during secondary recrystallization were investigated by the interrupted annealing method.

The precipitates were observed via transmission electron microscopy (TEM, JEM2100F, JEOL, Tokyo, Japan), and the chemical composition of precipitates was identified using a JEOL for energy dispersive spectroscopy (EDS) on the TEM. The orientation distribution functions (ODFs) of the deformed and annealed samples were examined at the quarter and center layers based on X-ray diffraction (Rigaku, SmartLab, Osaka, Japan). The microtexture was analyzed with a backscatter diffraction (EBSD) system with a scanning electron microscope (SEM, JEOL 6500F, Tokyo, Japan). The orientation distribution functions (ODFs) based on EBSD and X-ray diffraction were calculated by the series expansion method using the Bunge notation [20]. Moreover, the magnetostriction coefficients at room temperature under the applied fields from 0 to 1200 Oe were measured by using strain gages along the rolling direction (RD). The magnetostriction coefficient was calculated by the relation (3/2)λ = λ_//_ − λ_⊥_; λ_//_ and λ_⊥_ were the maximum magnetostriction values when the applied magnetic field was parallel and perpendicular, respectively.

## 3. Results

### 3.1. Microstructure and Texture of Rolling and Primary Recrystallization

**Figure 1:**
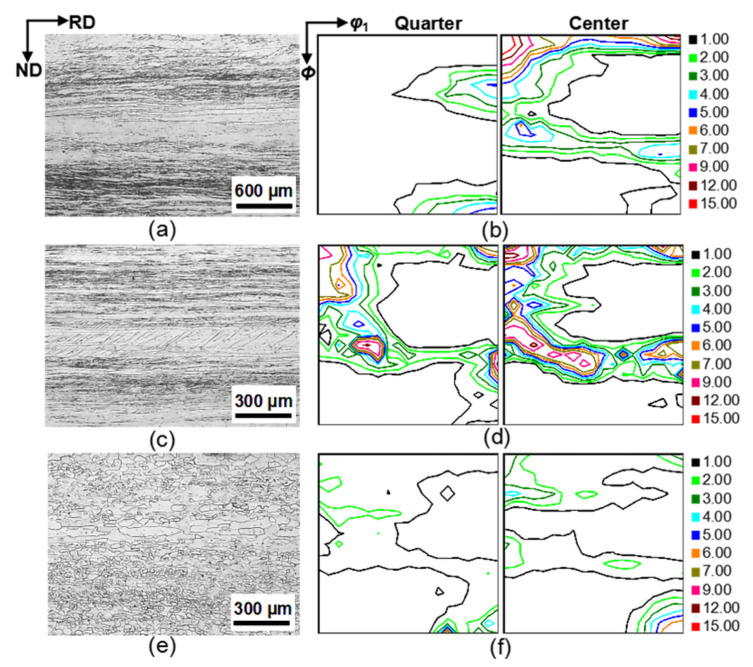
Microstructure and φ_2_ = 45° ODF sections in (**a**,**b**) hot-rolled strips, (**c**,**d**) first-stage cold-rolled sheets, and (**e**,**f**) intermediate annealed sheets of the Fe_81_Ga_19_ alloy.

**Figure 2:**
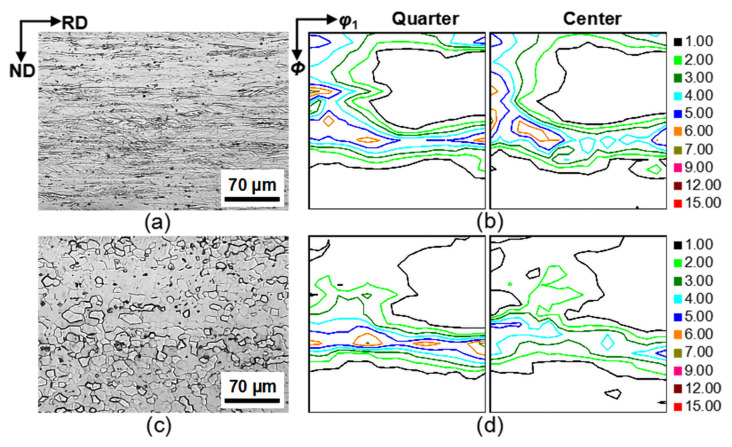
Microstructure (**a**,**c**) and φ_2_ = 45° sections of ODFs (**b**,**d**) in (**a**,**b**) second-stage cold-rolled sheets and (**c**,**d**) primarily recrystallized annealed Fe_81_Ga_19_ sheets.

Figure 1 and Figure 2 show the microstructure and texture at different processing stages. The hot-rolled strips were composed of the small grains at the subsurface layer (quarter) and elongated grains at the center layer (Figure 1a). The hot-rolled texture was characterized by Goss texture at the quarter layer, strong α fibers (<110>//RD) with a peak at {001} <110>, and weak γ fibers (<111>//ND) at the center layer (Figure 1b). The microstructure of the first-stage cold-rolled sheet was characterized by elongated grains with many in-grain shear bands through the sheet thickness (Figure 1c). The first-stage cold-rolled texture mainly comprised strong α fibers with a peak at {001} <110> and γ fibers with a peak at {111} <112> through the sheet thickness (Figure 1d). The intermediate annealed sheet was nearly completely recrystallized, with an average grain size of 21 μm and 36 μm at the quarter layer and the center layer (Figure 1e), respectively. The intermediate annealing texture mainly consisted of Goss and γ textures, where the Goss texture was stronger at the center layer (Figure 1f).

The second-stage cold-rolled sheets were composed of elongated deformed grains with a few in-grain shear bands (Figure 2a), and the texture had strong {223} <110> and {111} <112> components through the thickness (Figure 2b). The primarily recrystallized sheets were characterized by uniform and finer grains (~10 μm) through the thickness, and were textured by strong γ fibers (concentrated on {111} <112>) and weak α fibers (Figure 2c,d).

**Figure 3:**
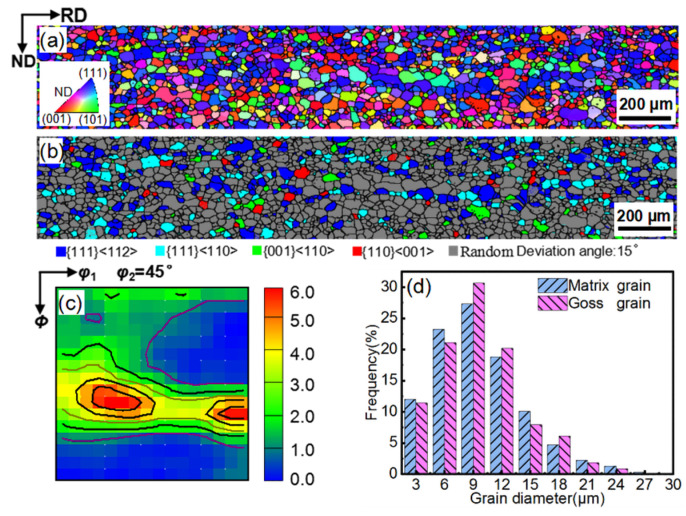
(**a**) Orientation image maps of all orientations, (**b**) several main texture components, (**c**) constant φ_2_ = 45°section of ODF, and (**d**) grain-size distribution of all grains and Goss grains in the primary recrystallized Fe_81_Ga_19_ thin sheet.

The orientation image maps of the primarily recrystallized sheets are presented in Figure 3. As shown, primary recrystallization was textured by the strongest {111} <112> texture (area fraction of 14.8%) and weak Goss texture (area fraction of 1.8%) through the thickness (Figure 3a,b). The Goss grains did not exhibit any advantage in terms of the grain size and quantity over the matrix grains (Figure 3d).

### 3.2. Inhibitor Evolution during Rolling and Annealing

**Figure 4:**
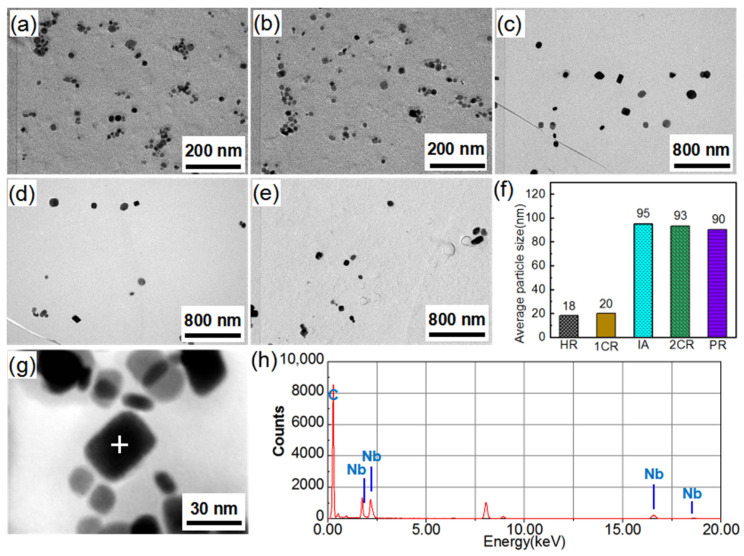
TEM micrographs of precipitates in the Fe_81_Ga_19_ thin sheet under different stages: (**a**) HR, (**b**) 1CR, (**c**) IA, (**d**) 2CR, and (**e**) PR. (**f**) The size distribution for different processes. (**g**,**h**) EDS of precipitates in the hot-rolled sheets.

Figure 4 presents the TEM micrographs and EDS data of the NbC precipitates in the Fe_81_Ga_19_ thin sheet during the rolling and annealing processes. Many finely dispersed spherical and rectangular precipitates with a size of ~18 nm and ~20 nm were formed in the hot rolling and first stages of the cold rolling of sheets. These precipitates were determined as NbC by EDS analysis, and were formed with a high density because of dislocation by a large reduction in the hot-rolled sheets (Figure 4g,h). After intermediate annealing, NbC precipitates grew up to a size of ~99 nm. After the second-stage cold rolling and primary recrystallization annealing, NbC precipitates dispersed with a diameter of ~90 nm. A small number of fine NbC particles were newly precipitated during primary recrystallization, resulting in a slight decrease in the size of the precipitates. The size of NbC precipitate was less than 100 nm, and a large quantity of NbC precipitates can provide a sufficient inhibition effect for primary recrystallization. This was confirmed by the observation that the average grain size was only 10 μm after primary recrystallization under this inhibitor, thereby providing suitable primary matrix grains for the secondary recrystallization.

**Figure 5:**
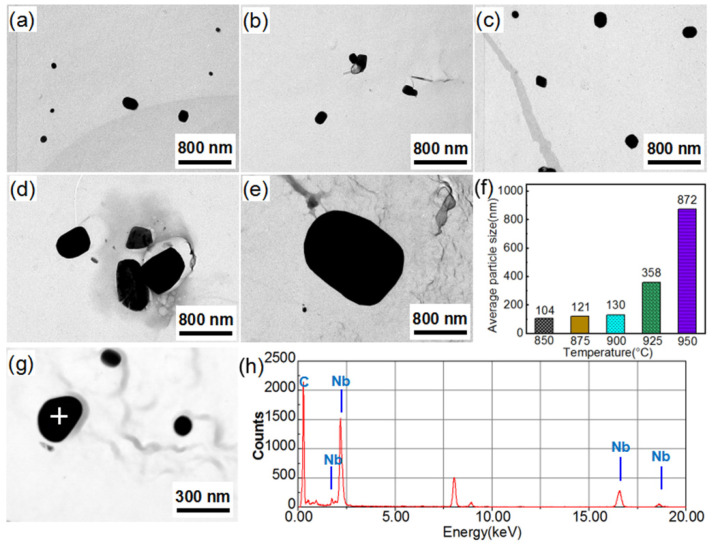
TEM micrographs of precipitates in annealed the Fe_81_Ga_19_ thin sheet with increasing temperature: (**a**) 850 °C, (**b**) 875 °C, (**c**) 900 °C, (**d**) 925 °C, and (**e**) 950 °C. (**f**) The size distribution at different temperatures. (**g**,**h**) EDS of precipitates at 850 °C.

Figure 5 shows the characteristics of precipitates during the high-temperature annealing process. With increasing temperature from 850 °C to 900 °C, the NbC precipitates slowly grew from a size of ~104 nm to ~130 nm. This slow coarsening of the NbC led to a gradual decrease in the pinning force acting on the matrix grains. The particles also were identified as NbC in the sheets extracted at 850 °C (Figure 5g,h). It was previously reported that the Goss grains in the primary recrystallization were surrounded by a high fraction of high-energy grain boundaries (HEGBs) [21,22,23], which exhibited higher mobility and facilitated the preferred growth for Goss grains under the same inhibiting force. When the inhibiting force slowly decreased, the driving force for the Goss grains could enhance and create a condition for preferred growth under lower temperatures. As the annealing temperature exceeded 925 °C, the precipitates rapidly grew to ~358 nm. The rapid coarsening of the NbC precipitates could reduce the pinning applied to the magnetic domain wall, and was conducive to the magnetic property of the Fe–Ga sheets [24].

### 3.3. Microstructure and Magnetostriction Evolution during High-Temperature Annealing

**Figure 6:**
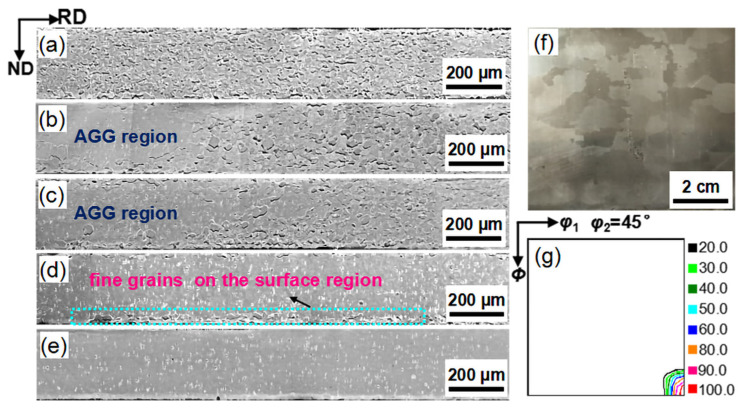
Microstructure evolution with increasing final annealing temperature to: (**a**) 825 °C, (**b**) 850 °C, (**c**) 875 °C, (**d**) 900 °C, and (**e**) 925 °C, as well as the (**f**) macrostructure and (**g**) constant φ_2_ = 45° section of ODF after annealing at 950 °C.

Figure 6 presents the microstructure evolution of the Fe_81_Ga_19_ sheets along the longitudinal section during secondary recrystallization annealing realized by the interrupted annealing method. Uniform grains with an average grain size of 11 μm were observed at 825 °C. This indicated that the matrix grains were effectively inhibited by the nanosized NbC precipitates. Abnormal grains with a size of several millimeters were observed as the temperature rose to 850 °C, but the matrix grains still had an average size of 12 μm. As the annealing temperature reached up to 900 °C, the secondary recrystallization grains occupied an area fraction of 85%, and colonies with fine grains sized ~15 µm were located at the surface of the sheet (as indicated by the rectangle in Figure 6d). This indicated that the slow coarsening of the NbC precipitates from 90 nm to 139 nm still provided an effective pinning force for the matrix grains. Secondary recrystallization was nearly completed at 925 °C. Complete secondary recrystallization with large grains of sizes ranging from 6 to 15 mm oriented by the Goss texture was achieved at 950 °C (Figure 6f,g).

**Figure 7:**
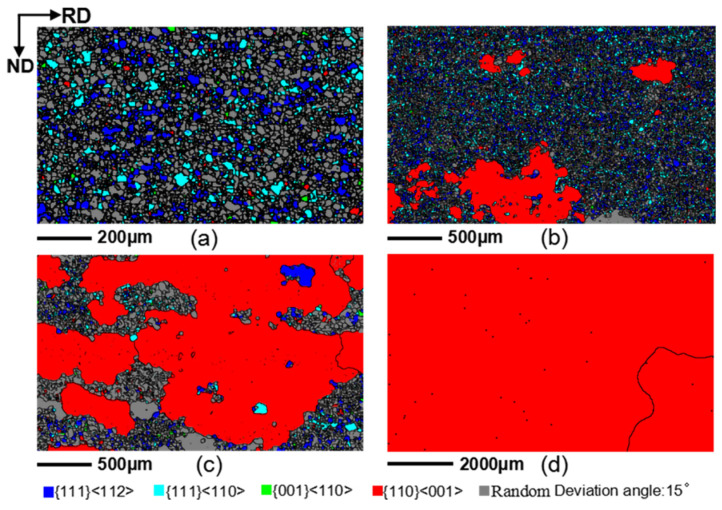
Orientation image maps of Fe_81_Ga_19_ thin sheets along the surface section heated to different annealing temperatures: (**a**) 825 °C, (**b**) 850 °C, (**c**) 900 °C, and (**d**) 950 °C.

Figure 7 shows the orientation image maps of Fe_81_Ga_19_ sheets along the surface section heated to different annealing temperatures. Before the onset of the secondary recrystallization, Goss grains only occupied an area fraction of 0.87% at 825 °C. The abnormally grown grains sized 0.2–1.0 mm at 850 °C were identified as Goss grains, and they occupied an area fraction of 15%. As annealing temperature increased to 900 °C, the secondary recrystallization Goss grains with a size of 0.5–4 mm formed rapidly, occupying an area fraction of 70%, and the matrix grain size was 15 µm. When the annealing temperature increased up to 950 °C, the area fraction of secondary recrystallization Goss grains with a size of 10 mm exceeded 99.8%, and the average deviation angle of the Goss grains was about 6°. The onset temperature (850 °C) and completion temperature (950 °C) of the secondary recrystallization of the Goss texture were much lower than those for the Fe–Ga thin sheets with microsized NbC particles and nanosized NbC or sulfide. Therefore, rapid secondary recrystallization at low temperatures was achieved in the present study by traditional rolling and annealing of the Fe–Ga thin sheet without applying any special atmosphere.

**Figure 8:**
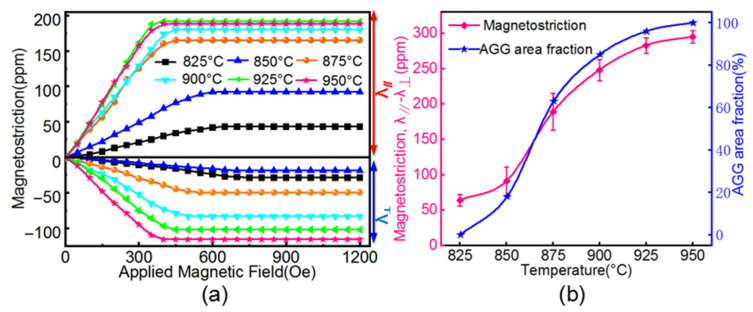
(**a**) Magnetostriction curves (λ_//_, λ_⊥_) against with magnetic field and (**b**) magnetostriction coefficients as a function of annealing temperature of the Fe81Ga19 thin sheet.

Figure 8 shows the measured magnetostriction coefficient of the Fe_81_Ga_19_ sheets under different annealing temperatures. The saturated magnetostriction coefficient clearly increased with increasing annealing temperature, and was consistent with variation of the area fractions of AGG Goss grains with increasing temperature. The occurrence of secondary recrystallization at 850 °C led to a magnetostriction coefficient of 91 ± 20 ppm; in contrast, the coefficient was 62 ± 7 ppm at 825 °C when no abnormal grains were formed. When the temperature approached 950 °C, a large magnetostriction coefficient of 295 ± 9 ppm was obtained in the Fe_81_Ga_19_ sheets, corresponding to sharp Goss orientation. This magnetostriction coefficient was higher than those reported for Fe–Ga alloys after secondary recrystallization by inhibitors and in a special atmosphere [11,12,13,14,15,16,17].

**Figure 9:**
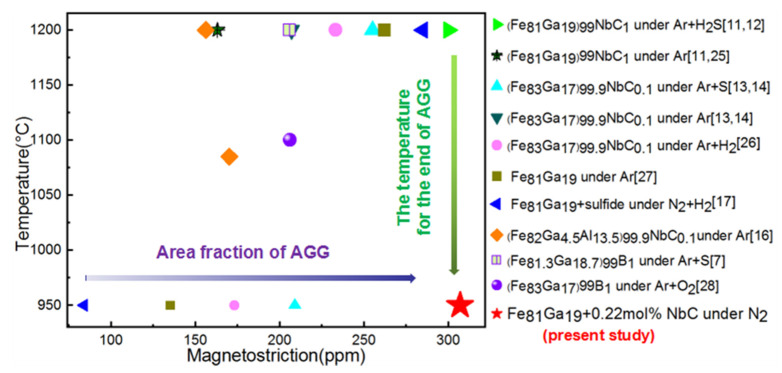
Final annealing temperatures and magnetostriction coefficients in Fe–Ga alloys corresponding to chemical compositions and annealing atmosphere.

Figure 9 summarizes the final annealing temperatures and magnetostriction coefficients of the Fe–Ga thin sheets as reported in the literature [7,11,12,13,14,16,17,25,26,27,28]. The magnetostriction coefficient of the Fe_81_Ga_19_ sheet secondarily recrystallization annealed in an N_2_ atmosphere in this study was equivalent to the values obtained in the cases of using microsized NbC particles and using the surface-energy effect of H_2_S; it was higher than the values obtained using nanosized sulfides in a N_2_ + H_2_ atmosphere, or using microsized NbC in Ar + S or H_2_ atmosphere; it was much higher than the values obtained for microsized Fe_2_B or NbC. Note that the final annealing temperature in this study was much lower than the values observed when using micro- or nanosized sulfides.

## 4. Discussion

The complete secondary recrystallization depends on the necessary inhibiting force for the normal grain growth of matrix grains and sufficient driving force for the AGG of Goss grains [29,30,31]. This driving force depends on the characteristics of the inhibitor, microstructure, and texture. In this work, the special inhibitors and the beneficial primary recrystallized microstructure and texture were prepared by the conventional rolling and annealing method to induce rapid secondary recrystallization without using any special atmosphere.

### 4.1. Favorable Inhibitor Characteristics

A large number of microsized NbC particles act as a strong inhibitor for the Fe–Ga thin sheet, but the surface energy effect of H_2_S is the main driving force for secondary recrystallization at high temperatures ranging from 1200 to 1300 °C, because the texture formed in primary recrystallization contributes little to the AGG of the Goss texture [18,32]. The nanosized NbC particles rapidly coarsen at temperatures of 900–950 °C, resulting in a rapid decrease in the pinning force; the advantage of the large grain size of the special initial columnar crystals is also required for complete recrystallization [13,14]. Nanosized sulfides with a size of 30–50 nm provide a strong pinning force for inhibiting the growth of matrix grains; however, the pinning force is too strong at low annealing temperatures, owing to the initial small size and low ripening rate; therefore, a higher annealing temperature is required for complete recrystallization [17].

In the present study, a number of finer NbC particles (0.22 mol%) with a size of ~18 nm were precipitated during the hot-rolling process (Figure 4a). After intermediate annealing and primary recrystallization, the size of the NbC precipitates increased to 90 nm (Figure 4b–e). The size of the NbC precipitates was relatively stable with increasing annealing temperature (<900 °C), and thus could provide enough pinning force for the matrix grains, and also provided enough driving force for priority growth of Goss grains, as shown in Figure 4, Figure 5 and Figure 6. Therefore, this special inhibitor characteristic allowed for controlling and sustaining a sufficient driving force for the rapid AGG of the Goss texture at low temperatures (≤950 °C) [33].

### 4.2. Matching Inhibitor Characteristics with Primary Recrystallization

Primary recrystallization microstructure and texture play a key role in determining the process of secondary recrystallization. In particular, the primary recrystallization was characterized by the presence of homogeneous fine grains (~10 μm) through the thickness and a narrow size distribution, as shown in Figure 3. These features were distinct from the primary recrystallization characteristics reported in the Fe–Ga sheet for high annealing temperatures with micro- or nanosized particles [11,12,13,14]. It was observed that the smaller grains with an average grain size of 30 μm occurred in the intermediately annealed sheets; the presence of these smaller grains was related to the large number of NbC precipitates with a size of 20 nm after the first-stage cold rolling. The high density of dislocations corresponding to the cold-rolling reduction of 75% provided abundant nucleation sites, and the high quantity of NbC precipitates with a size of ~90 nm inhibited the growth of recrystallized nuclei annealed at a temperature of 850 °C. This special microstructure provided the favorable characteristics of primary recrystallization for rapid secondary recrystallization.

In this study, the Goss texture did not exhibit any advantage in terms of the grain size and quantity over the matrix grains of the primarily recrystallized sheets (Figure 3d). However, the through-thickness strong {111} <112> and weak Goss textures could facilitate the abnormal growth of Goss grains by favoring high grain-boundary mobility from HEGBs [21,22,23]. The HEGBs around the Goss grains were considered to exhibit a higher mobility than that observed at the low-energy grain boundaries (LEGBs); this difference resulted in the preferential ripening of precipitates around the Goss grains. The mobility difference between the HEGBs and LEGBs was sensitive to the variations in the pinning force during the heating process. Therefore, it could be inferred that Goss grains used the advantage of the HEGBs to abnormally grow rapidly under decreasing pinning force at a low temperature. In the present study, owing to the regulation of the instability and intensity of the inhibitors and the favorable primary recrystallization, a sharp Goss texture with a large magnetostriction coefficient was achieved by rapid secondary recrystallization at a low temperature without the application of a special atmosphere

## 5. Conclusions

Herein, Fe_81_Ga_19_ + 0.22 mol% NbC sheets with a sharp Goss texture were successfully produced by a simple and efficient conventional rolling and annealing method induced by nanosized NbC precipitates. The conclusions are summarized as follows:

1. A higher quantity of nanosized NbC precipitates with a size of ~90 nm was prepared by hot rolling, first-stage cold rolling, intermediate annealing, and primary recrystallization annealing. The size of the NbC particles slowly increased to 130 nm as the temperature was increased to 900 °C in a pure nitrogen atmosphere.
2. Homogeneous fine grains (~10 μm) through the thickness were obtained after primary recrystallization annealing. The small grain size and narrower size distribution, as well as the strong γ fibers (concentrated on {111} <112>) and weak Goss texture in primary recrystallization, favored the rapid secondary recrystallization of the Goss texture.
3. The good matching of the special inhibitor characteristics and favorable primary recrystallization texture guaranteed the rapid secondary recrystallization of the Goss texture at annealing temperatures lower than 950 °C and resulted in a magnetostriction coefficient as high as 304 ppm in the Fe_81_Ga_19_ sheet.

## Data Availability

Data is contained within the article.

## References

[1] Clark A.E., Restor J.B., Wun-Fogle M., Lograsso T.A., Schlagel D.L. (2000). Magnetostrictive properties of body-centered cubic Fe-Ga and Fe-Ga-Al alloys. IEEE Trans. Magn..

[2] Guruswamy S., Srisukhumbowornchai N., Clark A.E., Restor J.B., Wun-Fogle M. (2000). Strong, ductile, and low-field-magnetostrictive alloys based on Fe-Ga. Scr. Mater..

[3] Kellogg R.A., Russell A.M., Lograsso T.A., Flatau A.B., Clark A.E., Wun-Fogle M. (2004). Tensile properties of magnetostrictive iron-gallium alloys. Acta Mater..

[4] Srisukhumbowornchai N., Guruswamy S. (2001). Large magnetostriction in directionally solidified FeGa and FeGaAl alloys. J. Appl. Phys..

[5] Kellogg R.A., Flatau A.B., Clark A.E., Wun-Fogle M., Lograsso T.A. (2003). Texture and grain morphology dependencies of saturation magnetostriction in rolled polycrystalline Fe_83_Ga_17_. J. Appl. Phys..

[6] Domann J.P., Loeffler C.M., Martin B.E., Carman G.P. (2018). High strain-rate magnetoelasticity in Galfenol. J. Appl. Phys..

[7] Na S.M., Flatau A.B. (2007). Secondary recrystallization, crystallographic texture and magnetostriction in rolled Fe-Ga based alloys. J. Appl. Phys..

[8] Na S.M., Yoo J.H., Flatau A.B. (2009). Abnormal (110) grain growth and magnetostriction in recrystallized galfenol with dispersed niobium carbide. IEEE Trans. Magn..

[9] He Z.H., Sha Y.H., Fu Q., Lei F., Jin B.K., Zhang F., Zuo L. (2016). Sharp Goss texture and magnetostriction in binary Fe_81_Ga_19_ sheets. J. Magn. Magn. Mater..

[10] Fu Q., Sha Y.H., He Z.H., Lei F., Zhang F., Zuo L. (2017). Recrystallization texture and magnetostriction in binary Fe_81_Ga_19_ sheets. Acta Metall. Sin..

[11] Na S.M., Flatau A.B. (2012). Single grain growth and large magnetostriction in secondarily recrystallized Fe-Ga thin sheet with sharp Goss (011)[100] orientation. Scr. Mater..

[12] Na S.M., Atwater K.M., Flatau A.B. (2015). Particle pinning force thresholds for promoting abnormal grain growth in magnetostrictive Fe-Ga alloy sheets. Scr. Mater..

[13] Yuan C., Li J.H., Zhang W.L., Bao X.Q., Gao X.X. (2015). Sharp Goss orientation and large magnetostriction in the rolled columnar-grained Fe-Ga alloys. J. Magn. Magn. Mater..

[14] Yuan C., Li J.H., Zhang W.L., Bao X.Q., Gao X.X. (2015). Secondary recrystallization behavior in the rolled columnar-grained Fe-Ga alloys. J. Magn. Magn. Mater..

[15] Liu Y.Y., Li J.H., Mu X., Bao X.Q., Gao X.X. (2017). Strong NbC particle pinning for promoting abnormal growth of Goss grain in Fe_82_Ga_4.5_Al_13.5_ rolled sheets. J. Magn. Magn. Mater..

[16] Liu Y.Y., Li J.H., Gao X.X. (2017). Influence of intermediate annealing on abnormal Goss grain growth in the rolled columnar-grained Fe-Ga-Al alloys. J. Magn. Magn. Mater..

[17] He Z.H., Hao H.B., Sha Y.H., Li W.L., Zhang F., Zuo L. (2019). Sharp secondary recrystallization and large magnetostriction in Fe_81_Ga_19_ sheet induced by composite nanometer-sized inhibitors. J. Magn. Magn. Mater..

[18] Na S.M., Flatau A.B. (2013). Global Goss grain growth and grain boundary characteristics in magnetostrictive galfenol sheets. Smart Mater. Struct..

[19] Hillert M. (1965). On the theory of normal and abnormal grain growth. Acta Metall..

[20] Bunge H.J., Esling C., Muller J. (1981). The influence of crystal and sample symmetries on the orientation distribution function of the crystallites in polycrystalline materials. Acta Cryst. A.

[21] Hayakawa Y., Szpunar J.A. (1996). The role of grain boundary character distribution in Goss texture development in electrical steels. J. Magn. Magn. Mater..

[22] Hayakawa Y., Szpunar J.A. (1997). A new model of Goss texture development during secondary recrystallization of electrical steel. Acta Mater..

[23] Rajmohan N., Szpunar J.A., Hayakawa Y. (1999). A role of fractions of mobile grain boundaries in secondary recrystallization of Fe–Si steels. Acta Mater..

[24] Yang F.Y., He C.X., Meng L., Ma G., Chen X., Mao W.M. (2017). Effect of annealing atmosphere on secondary recrystallization in thin-gauge grain-oriented silicon steel: Evolution of inhibitors. J. Magn. Magn. Mater..

[25] Chun H., Na S.M., Mudivarthi C., Flatau A.B. (2010). The role of misorientation and coincident site lattice boundaries in Goss-textured Galfenol rolled sheet. J. Appl. Phys..

[26] Yuan C., Li J.H., Bao X.Q., Gao X.X. (2014). Influence of annealing process on texture evolution and magnetostriction in rolled Fe–Ga based alloys. J. Magn. Magn. Mater..

[27] He Z.H., Sha Y.H., Fu Q., Lei F., Jin B.K., Zhang F., Zuo L. (2016). Secondary recrystallization and magnetostriction in binary Fe_81_Ga_19_ thin sheets. J. Appl. Phys..

[28] Li J.H., Gao X.X., Zhu J., Bao X.Q., Xia T., Zhang M.C. (2010). Ductility, texture and large magnetostriction of Fe–Ga-based sheets. Scr. Mater..

[29] Sakakura A. (1969). Effects of AlN on the primary recrystallization textures in cold-rolled-(110)[001]-oriented single crystals of 3% silicon Iron. J. Appl. Phys..

[30] Lin P., Palumbo G., Harase J. (1996). Coincidence site lattice (CSL) grain boundaries and Goss texture development in Fe–3% Si alloy. Acta Metall..

[31] Dennis J., Bate P.S., Humphreys F.J. (2009). Abnormal grain growth in Al–3.5Cu. Acta Metall..

[32] Na S.M., Flatau A.B. (2014). Texture evolution and probability distribution of Goss orientation in magnetostrictive Fe–Ga alloy sheets. J. Mater. Sci..

[33] Perez A.M., Dumont B.M., Acevedo-Reyes D. (2008). Implementation of classical nucleation and growth theories for precipitation. Acta Metall..

